# Clinical, economic, and humanistic burden of asthma in Canada: a systematic review

**DOI:** 10.1186/1471-2466-13-70

**Published:** 2013-12-05

**Authors:** Afisi S Ismaila, Amyn P Sayani, Mihaela Marin, Zhen Su

**Affiliations:** 1 Medical Affairs, GlaxoSmithKline Canada, Mississauga, ON, Canada; 2 Department of Clinical Epidemiology and Biostatistics, McMaster University, Hamilton, Ontario, Canada; 3 Product Value Strategy Consulting, Optum, Burlington, Ontario, Canada; 4 Medical Affairs, Sanofi, Cambridge, MA, USA

**Keywords:** Asthma, Literature review, Burden of illness, Costs, Quality of life

## Abstract

**Background:**

Asthma, one of the most common chronic respiratory diseases, affects about 3 million Canadians. The objective of this study is to provide a comprehensive evaluation of the published literature that reports on the clinical, economic, and humanistic burden of asthma in Canada.

**Methods:**

A search of the PubMed, EMBASE, and EMCare databases was conducted to identify original research published between 2000 and 2011 on the burden of asthma in Canada. Controlled vocabulary with “asthma” as the main search concept was used. Searches were limited to articles written in English, involving human subjects and restricted to Canada. Articles were selected for inclusion based on predefined criteria like appropriate study design, disease state, and outcome measures. Key data elements, including year and type of research, number of study subjects, characteristics of study population, outcomes evaluated, results, and overall conclusions of the study, were abstracted and tabulated.

**Results:**

Thirty-three of the 570 articles identified by the clinical and economic burden literature searches and 14 of the 309 articles identified by the humanistic burden literature searches met the requirements for inclusion in this review. The included studies highlighted the significant clinical burden of asthma and show high rates of healthcare resource utilization among asthma patients (hospitalizations, ED, physician visits, and prescription medication use). The economic burden is also high, with direct costs ranging from an average annual cost of $366 to $647 per patient and a total annual population-level cost ranging from ~ $46 million in British Columbia to ~ $141 million in Ontario. Indirect costs due to time loss from work, productivity loss, and functional impairment increase the overall burden. Although there is limited research on the humanistic burden of asthma, studies show a high (31%-50%) prevalence of psychological distress and diminished QoL among asthma patients relative to subjects without asthma.

**Conclusions:**

As new therapies for asthma become available, economic evaluations and assessment of clinical and humanistic burden will become increasingly important. This report provides a comprehensive resource for health technology assessment that will assist decision making on asthma treatment selection and management guidelines in Canada.

## Background

Asthma, an inflammatory disorder of the airways [1], accounts for roughly 80% of cases of chronic respiratory disease in Canada [2]. It affects more than 3 million Canadians and roughly 235 million people worldwide [3,4]. According to Statistics Canada, 8.5% of the population aged 12 and older has been diagnosed with asthma [5]. Its prevalence in this country has been increasing over the last 20 years [3]. Worldwide, asthma prevalence rates have been rising on average by 50% every decade [3]. Notably, asthma is the leading cause of hospital admissions in the overall Canadian population [3,6], the leading cause of absenteeism from school, and the third leading cause of work loss [3]. Each year, there are 146,000 emergency room visits due to asthma attacks in Canada [3]. Asthma is also a major cause of hospitalization [7] among the estimated 13% of Canadian children who suffer from the disease [8].

High prevalence in conjunction with significant asthma-related morbidity leads to a heavy clinico-economic and humanistic burden of asthma in Canada [9,10]. Healthcare utilization and costs are even higher when management and control of the disease are suboptimal [11]. The direct and indirect costs associated with asthma are expected to rank among the highest for chronic diseases due to the significant healthcare utilization associated with the disease [9] and asthma’s detrimental impact on physical, emotional, social, and professional lives of sufferers [12].

This systematic review is the first to consolidate and summarize the literature (from 2000–2011) encompassing not only the clinical and economic, but also the humanistic burden of asthma in Canada. It, thus, provides a holistic overview of the weight this disease poses to the healthcare system, patients and society. Specifically, this systematic literature review unveils the direct and indirect costs of asthma per patient, the key drivers of healthcare resource utilization, and the humanistic impact of asthma on patients’ quality of life (QoL), which cannot be inferred from clinical measures [13]. This information, consolidated in a single review, can be of value to payers, policy makers and healthcare providers in making decisions pertaining to the management and treatment of asthma.

## Methods

We conducted a search of the PubMed, EMBASE, and EMCare databases to identify original research (cross-sectional, observational, or longitudinal studies on the burden-of-illness and cost-of-illness) published from 2000 to 2011 on the burden of asthma in Canada. Review articles, letters, editorials, commentaries, studies reporting summaries of meeting proceedings or conferences, abstracts or posters presented at scientific meetings, and studies assessing the efficacy or effectiveness of specific interventions were not included. The time frame was selected to reflect more recent developments in the treatment and management of asthma in Canada.

Each search was conducted using controlled vocabulary and key words, with “asthma” as the main search concept. Search terms included “Canada,” “cost of illness,” “hospitalization,” “utilization,” “burden of illness,” “quality of life,” “sickness impact profile,” and “healthcare cost.” Appendix shows the detailed search strategies for each topic area. Searches were limited to articles published in English and studies involving humans. Studies were restricted to Canada.

Titles and abstracts of articles identified were carefully screened in the initial review for relevance to the topic. At the second review, articles were selected for inclusion based on predefined acceptance criteria, which included relevant patient population (ie, adults/children diagnosed with asthma) and appropriate study design and outcome measures (patient- and population-level). Two independent reviewers determined whether studies met the inclusion criteria, and discrepancies between reviewer decisions were resolved in consensus.

Reasons for study exclusions were recorded. For articles that met predefined inclusion/exclusion criteria, the quality of the studies was assessed using methodological checklists provided in the NICE Guidelines Manual [14] and the STROBE (STrengthening the Reporting of OBservational studies in Epidemiology) guidelines [15,16]. Key data elements were abstracted and tabulated in summary tables: year and type of study, number of study subjects, asthma definition, characteristics of study population, outcomes evaluated, results, and overall conclusions of the study.

Reported costs were inflated to 2011 Canadian dollars (CAD) using the Consumer Price Index from Statistics Canada [17].

## Results

**Figure 1:**
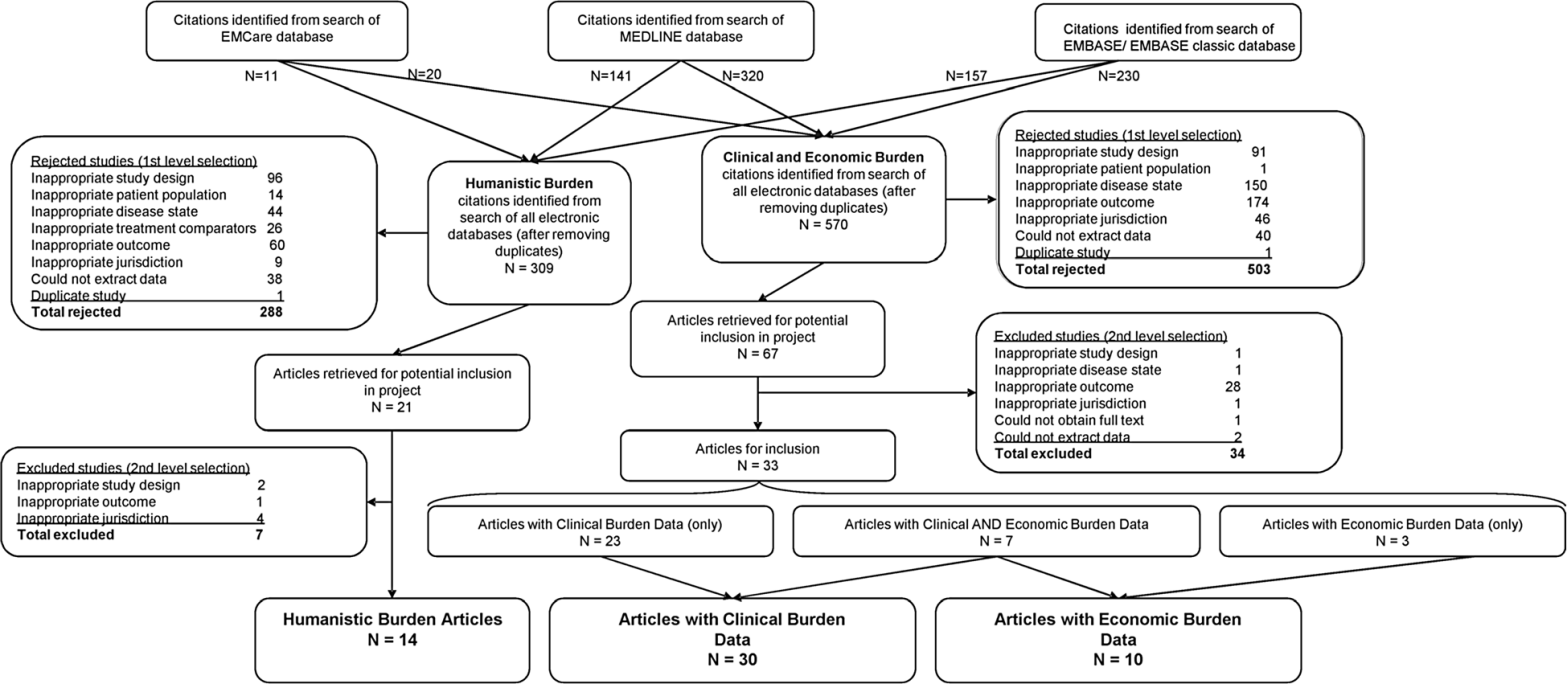
Process for studies to be included in the review.

Figure 1 depicts the step-by-step study selection process. The MEDLINE, EMBASE, and EMCare database searches yielded 320 citations, 230 citations, and 20 citations, respectively.

In the first-level selection process (based on the information presented in the article abstracts) for the clinico-economic burden, 503 of the 570 citations were rejected: 174 reported inappropriate outcomes (i.e., outcomes that were not aligned with the outcomes of interest), 150 due to inappropriate disease state (eg, the studies focused on other chronic respiratory diseases or included only a small number of the subjects with asthma), and 91 due to inappropriate study design. Other reasons for rejection during the first-level selection process are shown in Figure 1. Of the 67 full-text articles retrieved for potential inclusion, 34 were excluded during the second-level selection process (28 due to inappropriate outcomes). Thus, 33 articles fulfilled all criteria and were included in the clinico-economic burden review (Figure 1).

After duplicates were removed, 309 studies were identified by the humanistic burden literature searches from the 3 databases. Of these, 288 studies were excluded during the first-level selection for inappropriate disease state (n = 44), inappropriate outcome measure (n = 60), inappropriate study design (n = 96), jurisdiction (n = 9), inappropriate patient population (n = 14), treatment comparator (n = 26), because data could not be extracted in the required format (n = 38), or because they were duplicate studies (n = 1).Twenty-one studies were selected for potential inclusion in the review. During the second-level selection, full-text articles were reviewed and a further 7 were excluded for inappropriate outcome measure (n = 1), study design (n = 2) or jurisdiction (n = 4). Fourteen articles fulfilled all criteria and were included in the humanistic burden review (Figure 1).

Table 1 depicts the quality assessment of the articles on clinical, economic, and humanistic burden using STROBE tools, and Table 2 summarizes quality assessment of the articles on clinical burden using the NICE RCT assessment tool.

**Table 1:** Summary of quality assessment (using STROBE assessment tools) of the articles included

**Report section**	**Item**	**Item #**	**% articles with STROBE criteria not met**		
			**Clinical burden**	**Economic burden**	**Humanistic burden**
**Title and abstract**	**Title**	1a	20%	40%	10%
	**Abstract**	1b	13%	30%	0%
**Introduction**	**Background/rationale**	2	0%	0%	0%
	**Objective**	3	0%	0%	0%
**Methods**	**Study design**	4	3%	10%	0%
	**Setting**	5	0%	0%	0%
	**Participants**	6a	13%	10%	0%
		6b	30%	10%	10%
	**Variables**	7	23%	30%	30%
	**Data sources/measurement**	8	10%	10%	0%
	**Bias**	9	53%	40%	40%
	**Study size**	10	20%	30%	30%
	**Quantitative variables**	11	13%	20%	20%
	**Statistical methods**	12a	30%	30%	10%
		12b	47%	60%	20%
		12c	70%	60%	50%
		12d	60%	60%	30%
		12e	77%	50%	80%
**Results**	**Participants**	13a	40%	50%	30%
		13b	63%	60%	30%
		13c	73%	80%	50%
	**Descriptive data**	14a	37%	50%	20%
		14b	77%	80%	60%
		14c	27%	20%	20%
	**Outcome data**	15	3%	10%	0%
	**Main results**	16a	27%	30%	0%
		16b	63%	70%	20%
		16c	40%	70%	30%
	**Other analyses**	17	37%	20%	60%
**Discussion**	**Key results**	18	0%	0%	0%
	**Limitations**	19	7%	20%	20%
	**Interpretation**	20	3%	10%	0%
	**Generalizability**	21	3%	10%	20%
**Other**	**Funding**	22	23%	40%	20%

**Table 2:** Summary of quality assessment (using NICE RCT assessment tool) of the articles included

**Type of bias**	**Humanistic burden (n=4)**	
	**Low risk**	**Unclear risk**
Selection	2	2
Performance	3	1
Attrition	2	2
Detection	3	1

### Clinical burden studies

#### ***Overview***

Of the 33 studies meeting all criteria for inclusion, 23 contained clinical burden data *only*, 7 had information on both clinical and economic burden of asthma, and 3 had data on the economic burden of asthma only.

Of the 30 studies on clinical burden, 1 was a case–control, 22 were cohort, and 7 were cross-sectional studies. Characteristics of studies reporting on clinical burden are shown in Table 3.

**Table 3:** Characteristics of clinical burden studies included in the review

**Reference/Study period**	**Data source**	**Study objective**	**Inclusion criteria**	**Asthma definition**
***Retrospective cohort studies***				
Sadatsafavi et al. 2010 [10] 1996 - 2000	Administrative healthcare data	Determine direct medical costs of asthma-related healthcare in British Columbia	5 to 55 years	*Narrow:* ICD-9 493.x *Broad*: visits for an asthma-related diagnosis; hospitalizations with asthma among the discharge diagnoses
			≥4 asthma prescriptions in 1 year	
			≥1 asthma hospitalization	
			≥2 physician visits for asthma	
Blais et al. 2011 [18] 1998 - 2005	RAMQ database,	Determine relationship between better use of LTRA and asthma exacerbations in children	5-15 years	Moderate or severe asthma exacerbations - an ED visit for asthma, a hospital admission for asthma, or a dispensed short-course (14 days) prescription of oral corticosteroids
			Diagnosed asthma	
			Initiating (mono)therapy with ICS or LTRA	
Rosychuk et al. 2010 [19] Apr 1999 to Mar 2005	Provincial administrative healthcare databases	Describe the epidemiology of asthma presentations to EDs for 3 main regions in the province of Alberta	All people registered under the AHCIP at any time in a given year	ICD-9 code 493.x or ICD-10 code J45.x as the first or second diagnosis fields in the ACCS
Crighton et al. 2001 [20] Apr 1, 1988 to Mar 31, 2000	DAD database at CIHI,	Examine the seasonal patterns and trends of asthma hospitalizations in relation to age and gender	NR	ICD-9-CM code 493
Ungar et al. 2011 [21] Nov 1, 2000 to Mar 31, 2003	Interview data linked to administrative healthcare data.	Identify factors associated with asthma exacerbation causing ED visits or hospitalizations related to health status, socioeconomic status (SES), and drug insurance	1 to 18 years	Physician-diagnosed asthma; ICD-9 493 or ICD-10 J45
Disano et al. 2010 [22] 2003 - 2006	DAD database from CIHI, INSQP Deprivation Index, Statistics Canada Community Profiles	Examine inequalities between SES groups with respect to rates of ACSC-hospitalizations	Acute care cases of 0 to 75 years; asthma in children for age <20 years	NR
Blais et al. 2009 [18] 2002 - 2004	RAMQ database	Compare the use of healthcare services between new users of budesonide/formoterol and F/S	Asthma patients aged 16 to 65 years ≥1 claim for combination therapy in 2002 or 2003 and no claims for combination therapy for ≥1 year prior to first claim	ICD-9 codes 493.0, 493.1, 493.9
Rowe et al. 2009 [23] 1 Apr 1999–31 Mar 2005	ACCS and other provincial databases.	Describe the epidemiology of asthma presentations to EDs made by adults in the province of Alberta, Canada	Asthmatic individuals aged 18 years	ICD-9 493.x or ICD-10 J45.x
To et al. 2008 [24] 1994 - 1998	DAD database from CIHI, OHIP records, RPDB database	Describe the prevalence of asthma; all-cause mortality; physician visits and hospitalizations for asthma and all causes; and seasonal and geographical variation of healthcare utilization in children	Children aged 0 to 9 years	At least 1 asthma hospitalization or 2 asthma OHIP claims within 3 years
Lemiere et al. 2007 [25] 2001 - 2004	RAMQ database, WRA patients	Compare clinical characteristics and use of medical resources between subjects with OA, WEA, and WRA	NR	Physician-diagnosed asthma OA, WEA, and WRA
To et al. 2007 [26] 1994 to 2006	HMDB database from CIHI, OHIP records, RPDB database;	Examine and predict the persistence of childhood asthma	Children born in 1994 diagnosed with asthma before their 6th birthday, followed up until their 12th birthday	1 asthma hospitalization or 2 asthma physician claims within 3 years prior to age 6 years (ICD-9 493 or ICD-10 J45). Persistent asthma - additional claims during follow-up Remission asthma - no additional claims
Agha et al. 2007 [27] 1993 - 2001	DAD database at CIHI, SES from the 1996 Census data	Examine socioeconomic disparities in ACS and non-ACS admissions among birth cohorts in a universal health insurance setting	Children born alive in Toronto during 1993–2001	The most responsible diagnosis in the CIHI DAD DB
Gershon et al. 2007 [2] 1994/95 to 2001/202	DAD from CIHI, OHIP	Understand the burden of asthma	Asthma patients from ON, aged 0–39 years	1 DAD hospitalization record or 2 OHIP claims for asthma in a 3-year period
Lougheed et al. 2006 [28] 2001 - 2002	CIHI	Assess regional differences in ED visit rates and hospitalizations for asthma	ED visits for asthma	ICD-10 code J45.x
Dik et al. 2006 [29] 1985 - 1998	Manitoba administrative healthcare data	Study 14-year trends in utilization of physician resources for asthma and compare them to trends for allergic rhinitis	NR	ICD-9-CM code 493
Sin et al. 2001 [30] FY 1992 - 1996	CIHI, drug claims, physician billing, and mortality databases	Determine the impact of ICS on rehospitalization for asthma and all-cause mortality rates in elderly patients	Asthmatic patients, aged ≥65 years, who had been hospitalized with a most responsible diagnosis of asthma in the past 5 years	ICD-9 codes 493.0, 493.1, and 493.9
***Prospective cohort studies***				
Rowe et al. 2010 [31] 2004 – 2005	Interviews	Describe factors associated with admission to hospital for acute asthma after ED treatment	Patients aged 18 to 55 years diagnosed with asthma	Patient-reported
Sin et al. 2003 [32] 1985, 1988	AHCIP data,	Determine the relationship between SES and ED visits for asthma in a free access healthcare system.	Children born 1985 to 1988 followed for 10 years	ICD-9 code 493.x
Ungar et al. 2001 [33] May - Oct 1995	Telephone interviews at 1, 3, and 6 months,	Assess the cost of asthma care at the patient level in children from the perspectives of society, the Ontario Ministry of Health, and the patient.	Patients or caregivers filling prescriptions for bronchial inhalers	Probable asthma - a prescription for a bronchial inhaler medication in the last month (bronchodilator or corticosteroid) and reported experiencing shortness of breath, wheeze, or recurrent cough in the past
Anis et al. 2000 [34] Sept 1, 1994 - Aug 31 1995	Hospital ED, telephone interview for follow-up	Estimate the average direct cost of illness for 4 cardiorespiratory conditions	ED visitors who completed follow-up interviews	ED visit records
Rowe et al. 2007 [23] 1996-1998	Structured ED interview and telephone follow-up 2 weeks later	Compare ED asthma management and outcomesbetween Canada and US	Patients aged 2 to 54 years who presented with acute asthma in ED	NR
***Cross-sectional studies***				
Boulet et al. 2008 [35] April - August 2004,	Telephone survey	Assess the influence of current and former smoking on self-reported asthma control and healthcare use	Adults aged 18 to 54 years with physician-diagnosed asthma for ≥6 months	Patient-reported or physician-diagnosed asthma
Klomp et al. 2008 [36] 2002/03 and 2003/04	Health databases in Saskatchewan	Describe the quality of asthma care using a set of proposed quality indicators	Saskatchewan residents who had a valid health insurance number	Over 1-year period: ≥3 prescriptions for antiasthma drug or ≥2 physician claims (ICD-9 code 493) or ≥2 hospitalization claims (ICD-9 493.x or ICD-10 J45.x) or ≥1 claim for physician services or hospitalization for asthma plus ≥1 pharmacy claim for an antiasthma drug
Iron et al. 2003 [37] 1994/1995	CNPHS data, OHIP	Determine the association between demographics, access to care, SES, and need (comorbidities) with actual family physician costs	Survey respondents aged ≥25 years consenting to share HC# and responses with MOHLTC	Self-reported
Anis et al. 2001 [38] 1995	Ministry of Health administrative databases	Determine whether excessive use of SABA, in conjunction with underuse of ICS, would be a marker for poorly controlled asthma and excessive use of healthcare resources	Asthma patients aged 5 to 50 years for whom ≥1 prescription for a SABA was filled in 1995	Patients filling SABA prescriptions; for hospitalizations, ICD-9 code 08 (diseases of the respiratory system)
Baibergenova et al. 2005 [39] April 1, 2001 to March 31, 2004		Examine the pattern and strength of seasonal fluctuations in ED visits due to asthma	Asthma patients with ED visits for asthma or status asthmaticus	ICD-9 code 493.x or ICD-10 J45.0–J45.9
Lynd et al. 2004 [40] NR	Survey	Assess the association between SES and SABA use, controlling for asthma severity	Asthmatic patients aged 19 to 50 years residing in the Greater Vancouver Regional District of British Columbia	NR
***Case–control study***				
Suissa et al. 2002 [41] 1975 - 1997	Saskatchewan Health DB	Assess whether regular use of ICS prevents asthma hospitalizations	*Source cohort*: subjects aged 5–44 years receiving ≥3 prescriptions of an antiasthma medication in any 1-year period *Full cohort*: all subjects with ≥1 year follow-up, irrespective of whether they were admitted to hospital for asthma during the baseline year	Primary discharge diagnosis of asthma (ICD-9 codes 493.0, 493.1, or 493.9)
***Health economic analysis***				
Seung et al. 2005 [42] 2004	NACRS at CIHI, OCCI, MOHLTC billing	Determine the use of urgent care resources and annual costs for the uncontrolled asthmatic population in Canada	NR	ICD-9 Code 493

ACCS=ambulatory care classification system, ACSC=ambulatory care-sensitive conditions, AHCIP=Alberta Healthcare Insurance Plan, CIHI=Canadian Institute for Health Information, CNPHS=Canadian National Population Health Survey, DAD=Discharge Abstract Database, ED=emergency department, HMDB=Hospital Morbidity Database, ICS=inhaled corticosteroid, ICD=International Classification of Diseases, LTRA=leukotriene receptor antagonist, MOHLTC=Ministry of Health and Long Term Care, NACRS=National Ambulatory Care System, NR=not reported, OA= occupational asthma, OCCI=Ontario Case Costing Initiative, OHIP=Ontario Health Insurance Plan, RAMQ=Régie de l’assurance maladie du Québec, RPDB=Registered Persons Database, SES=socioeconomic status, WEA=work-exacerbated asthma, WRA=work-related asthma.

Most studies clearly reported the study design (97%), setting (100%), participants (87%), and statistical methods employed (70%). However, less than half reported on potential sources of bias and confounding factors or how missing data was handled. Furthermore, less than half of the studies reported on how loss to follow-up was addressed in both the methods and results sections, or how sensitivity analyses were conducted. Main results for outcomes data were appropriately reported in 97 % of the clinical burden studies, and more than 90% met the STROBE criteria for appropriate quality discussion. Most (77%) gave the source of study funding and the roles of the funders. (Tables 1 and 2).

Studies employed a variety of definitions for asthma, including ICD codes, physician visits and/or hospitalizations for asthma (based on billing codes), asthma medication prescriptions filled, and patient self-report. We report the definitions used, but these definitions were not reconciled in this review. When asthma was defined by the presence of ICD codes, it was considered to be narrowly defined, whereas a broad asthma definition included visits for an asthma-related diagnosis and asthma-related hospitalizations among the discharge diagnoses.

### Key findings on clinical burden

#### ***Hospitalizations***

Table 4 provides an overview of hospitalization rates for adult and pediatric patients with asthma in Canada. Reported rates of hospitalization for asthma varied widely according to age, geographic region, gender, and asthma medication use. In a large cohort study spanning over 20 years, Suissa et al. [41] obtained data from the Saskatchewan Health databases on asthma patients from that province aged 5–44 between 1975 and 1991 and found that the overall rate of asthma hospitalization was 42 per 1000 asthma patients per year in patients with at least 1 year of follow-up. The rate was higher (48 per 1000) in patients receiving at least 3 anti-asthma medication prescriptions in any 1 year. During the variable follow-up period (up to 4 years), regular use of inhaled corticosteroids (ICS) was associated with a 31% reduction in the rate of hospital admissions for asthma and a 39% reduction in the rate of readmissions for the cohort with more severe asthma who had been previously hospitalized for the condition during the 1-year baseline period. The study investigators concluded that their findings emphasize the importance of regular use of inhaled corticosteroids to avoid hospitalizations.

**Table 4:** Rate of hospitalizations for asthma patients in Canada

**Study**	**Number of patients**	**Patient descriptor**	**Year**	**Hospitalizations for asthma**	
				**Per patient per year**	**Per 1000 patients per year**
***Children***					
Blais et al. 2011 [43]	7,494	≥1 exacerbations in the year prior to treatment initiation, ICS	1998-2005	0.03	
		≥1 exacerbations in the year prior to treatment initiation, LTRA		0.06	
	19,861	No exacerbation in the year prior to treatment initiation: ICS		0.005	
		No exacerbation in the year prior to treatment initiation: LTRA		0.003	
Ungar et al. 2011 [21]	490	Asthmatic children	2000-2003	0.25§	
To et al. 2008 [24]	56,737	0-2 years	1998/1999		86.7
	99,163	3-5 years			27.3
	141,305	6-9 years			10.9
	297,205	Overall			30.9
To et al. 2007 [26]	34,216	Persistent asthma	1994-2006		63*
		Remission asthma			39*
		Overall			52*
Ungar et al. 2001 [33]	339	Asthma children	1995	1	
***Adults***					
Sadatsafavi et al. 2010 [10]	158,516	Narrow asthma definition€	1996-2000	0.016	
		Broad asthma definition¥		0.03	
Lemiere et al. 2007 [25]	351 (WEA: 145, OA: 206)	WRA	2001-2003	0.04(0.2)	
		NWRA		0.008(0.7)	
Anis et al. 2001 [38]	4,671	Appropriate use†	1995	0.07(0.34)	
	763	Inappropriate use‡		0.11(0.42)	
***All ages***					
Disano et al. 2010 [22]	NR	High SES	2003-2006		1.61**
		Average SES			1.95**
		Low SES			2.7**
Klomp et al. 2008 [36]	24,616 (24,180 of whom were still alive and living in the region the following year)	Asthma patients	2002/2003 and 2003/2004		10.9
Lougheed et al. 2006 [28]	574,304 children and 1,194,095 adults in Ontario	Patients with an ED disposition diagnosis of asthma in a stratified sample of 16 hospitals	2001-2002		108 (10.8%) children; 69 (6.9%) adults
Suissa et al. 2002 [41]	30,569	Source cohort††	1975-1997		48
	4,673	Full cohort‡‡			42.4
Seung et al. 2005 [42]	NR	Asthma patients	2004		1.43**

§Calculated as 124 hospitalizations for 490 patients.

*Calculated as the rate per 100 patients x 10.

**Calculated as (the rate per 100,000 patients) / 100.

€Narrow asthma definition: ICD-9 493.x.

¥Broad asthma definition: visits for an asthma-related diagnosis; hospitalizations with asthma among the discharge diagnoses.

†Appropriate use (low-dose SABA + high-dose ICS).

‡Inappropriate use (high-dose SABA + low-dose ICS).

††Source cohort: subjects 5–44 years receiving ≥3 prescriptions of an anti-asthma medication (beclomethasone, budesonide, epinephrine bitartrate, fenoterol, flunisolide, ipratropium bromide, isoproterenol, ketotifen, metaproterenol, nedocromil, procaterol, salbutamol, sodium cromoglycate, terbutaline, triamcinolone acetate, or any compound of theophylline) in any 1 year period.

‡‡Full cohort: all subjects with at least 1 year follow up, irrespective of whether or not they were admitted to hospital for asthma during the baseline year.

NWRA=non-work-related asthma; WRA=work-related asthma.

In a retrospective cross-sectional study of asthma patients aged 5–54 years using health databases in Saskatchewan, Klomp et al. [36] found that, in 2002–03 and 2003–04, the hospitalization rate for asthma was 10.9 per 1000 patients per year.

Agha et al. [27], using data on hospital admissions from the Dischrage Abstract Database of the Canadian Institute for Health Information, reported 8,583 asthma hospitalizations among 255,284 pediatric patients (a rate of 33.6 in 1000 patients) born between 1993 and 2000 in Toronto.

A significantly lower rate was reported in Canada by Seung et al. [42], who cited figures reported by the Public Health Agency of Canada of 143 asthma-related hospitalizations per 100,000 adult and pediatric patients, or 1.43 in 1000, in 1998 (with an additional 3.7 per 1000, many of whom had underlying asthma, hospitalized for influenza/pneumonia).

Higher rates were reported for hospital admissions of patients who initially presented to the emergency department (ED). Lougheed et al. [28] reported that 6.9% of adults and 10.8% of children who presented to the ED with asthma were admitted to the hospital.

According to the results of a study based on interviews with parents, 25% of the pediatric study population (124 of 490 patients) had been hospitalized for asthma in the previous 12 months [21]. In a large study utilizing data from Quebec administrative databases, children aged 5 to 15 years with at least 1 exacerbation in the year prior to treatment initiation with ICS or leukotriene receptor antagonists (LTRA) had higher rates of hospitalizations than those with no exacerbation in the previous year (0.03 vs. 0.005 hospitalizations per patient per year in the ICS group and 0.06 vs. 0.003 per patient per year in the LTRA group) [43]. The proportion of prescribed days covered was significantly higher in the LTRA group than in the ICS group (52% vs. 34%) [43].

In a study of all Ontario babies born during the year 1994 who were diagnosed with asthma before their sixth birthday, there was a decreasing trend in hospitalization rates with age, from 86.7 per 1000 patients per year in the 0 to 2 years age group to 27.3 per 1000 patients for those aged 3 to 5 years and 10.9 per 1000 for those aged 6 to 9 years. These investigators also found that children with persistent asthma had more than one and a half times higher hospitalization rates compared with patients whose asthma was in remission (63 per 1000 patients vs. 39 per 1000 patients per year) [26].

In another Ontario-based study that examined asthma seasonality and hospitalizations by gender and age group over a 12-year period, results of spectral analysis revealed that hospitalization rates for children with asthma were highest in September and October each year across the 12-year period, with a 2 to 3-times higher rate of hospitalizations in boys (180 per 100,000) than in girls under the age of 9 years [20]. However, among children older than 9 years, female hospitalizations exceeded those of males [20].

The large variations in reported rates of hospitalizations may be due to variations in ED visit rates and/or hospital admission percentages [28]. Hospital admissions appear to follow a bimodal age distribution pattern, with the very young and the elderly more likely to be admitted [28]. Other factors that can drive up rates of hospitalization in particular regions or among specific populations are higher disease prevalence, greater disease severity, multiple comorbidities, and barriers to care associated with socioeconomic status [27].

#### ***ED visits***

The number of asthma emergency visits varied by age, type of treatment, social status, and living area (urban/non-urban). Table 5 summarizes ranges and mean numbers of annual ED visits for asthma, as reported in the included studies. According to several studies, both children and adults with asthma averaged less than 1 ED visit per patient per year [21,23,28]. ED visit rates were significantly higher in women than in men and, overall, the rate of ED visits increased with age [28].

**Table 5:** Annual number of ED visits for asthma, per patient, in Canada

**Reference**	**Number of patients**	**Descriptor**	**Annual mean number (SD) of ED visits per patient for asthma (range)**	
			**From**	**To**
*Children*				
Blais et al. 2011 [43]	27,355	Children, 5–15 years, on ICS or LTRA therapy, by # of exacerbations in the previous year, 1998-2005	0.04* (on LTRA, no exacerb. in the previous year)	0.32* (on ICS, 1+ exacerb. in the previous year)
Lougheed et al. 2006 [28]	4,674	Ontario patients, <20 years, 2001-2002	13.6 [8.7 to 25.2]**	
Sin et al. 2003 [32]	90,845	Children, 0–10 years, 1985–1988, by SES	6 [0 to 31]** (very poor)	7 [0 to 34]** (non-poor)
Ungar et al. 2001 [33]	339	Children with asthma, <15 years, Ontario, 1995	0.8*	
*Adults*				
Sin et al. 2001 [44]		elderly asthmatic, by ICS therapy	1 (1.2)* (not using ICS)	1.5 (1.3)* (using ICS)
Rowe et al. 2009 [45]	48,942	Adults, 1999/2000 to 2004/2005	6.7** (2004/2005)	9.7** (1999/2000)
Lemiere et al. 2007 [25]	351	Adults, work-related asthma, 2001-2004	0.2 (0.7)* (NWRA)	0.3 (0.8)* (WRA)
Lougheed et al. 2006 [28]	3,993	Adults, ≥20 years, 2001-2002	3.9 [1.7 to 10.1]**	
Anis et al. 2001 [38]	5,434	Adults, use of SABA+ICS, 1995	0.04 (0.26)* (appropriate use∫)	0.08 (0.33)* (inappropriate use∫)
Rowe et al. 2007 [23]	3,031	Canada and US ED visits, 1996-1998	0(0–3)§ (US)	1(0–3)^§^ (Canada)
Baibergenova et al. 2005 [39]	73,566	Adult, Ontario, 2001-2004	0.45^†^	
*All ages*				
Rosychuk et al. 2010 [19]	21,700	Asthma patients, Alberta, 2004-2005	6.9(6.6-7.0)*** (Calgary)	15.1(15.1-15.9)*** (NMU)

*Per patient.

**Mean [range] per 1000 patients.

***Mean (95%CI).

§Median (IQR).

∫Appropriate use (low-dose SABA + high-dose ICS); Inappropriate use (high-dose SABA + low-dose ICS).

†Calculated from 99,054 ED visits due to asthma were made by 73,566 adults.

*ICS=inhaled corticosteroid, LTRA=leukotriene receptor antagonist, NMU= non-major urban areas, NWRA=non-work-related asthma, SABA=short-acting β-agonist, WRA=work-related asthma*.

In a study investigating the impact of appropriate use (according to the 1999 Canadian asthma consensus report and the National Heart, Lung and Blood Institute Guidelines for the Diagnosis and Management of Asthma) and compliance with asthma medications in adults, the rate of ED visits for asthma was twice as high for patients not using asthma medication appropriately (high-dose SABA plus low-dose ICS) than for those using it appropriately (low-dose SABA plus high-dose ICS) [38].

Rosychuk et al. [19] examined trends in asthma-related ED visits by more than 45,000 children aged <18 years during the period from April 1999 to March 2005 and did not observe decreased ED presentation rates over time, despite improvements in treatment and availability of guidelines. The standardized rates remained stable over time, with 21.1 visits occurring per 1000 patients in 1999/2000 versus 19.8 per 1000 in 2004/2005.

Sin et al. [30,44] reported that elderly asthmatic patients using ICS post-discharge from hospital were 29% less likely to be readmitted to hospital for asthma and 39% less likely to experience all-cause mortality compared with those who did not receive ICS post-discharge over a 1-year follow-up period. When age, sex, comorbidity, and use of other antiasthma medications were controlled for, ICS use was associated with a 32% relative rate reduction for recurrent hospitalization or all-cause mortality (95% CI 23%-39%). Among patients who received at least 1 prescription for ICS within 1 year prior to the index hospitalization, the use of ICS 90 days post-discharge was associated with a 41% decrease in recurrent asthma-related hospitalizations or deaths compared with non-use of ICS (95% CI 32%-49%).

Sin et al. [32] also reported on ED visits in children born in Alberta between 1985 and 1988, stratified by SES, and found that very poor children were 23% more likely to have had an ED visit for asthma compared with children from non-poor families (RR 1.23; 95% CI 1.14 – 1.33). Very poor children had a similar risk of having an asthma-related ED visit as poor children (RR 0.97; 95% CI 0.91 – 1.04).

#### ***Physician visits***

Studies that reported rates of asthma-related physician visits are summarized in Table 6. In a population-based study evaluating 14-year trends in Manitoba in utilization of physician resources for asthma, Dik et al. [29] found that, between the period 1985–1988 and 1994–1998, the greatest increases in prevalence and incidence of physician visits for asthma occurred in the youngest age groups, while in adults the prevalence and incidence changed little with time. However, the average rate of physician visits for asthma decreased from 1.66 visits per patient-year in 1985–1988 to 1.40 in 1989–1993, and further to 1.16 visits per patient-year in 1994–1998.

**Table 6:** Rate of physician visits in Canada

**Study**	**Number of patients**	**Patient descriptor**	**Year**	**Physician visits for asthma**^**§**^
***Children***				
To et al. 2008 [24]	56,737	0-2 years	1998/1999	2.2
	99,163	3-5 years		1.1
	141,305	6-9 years		0.8
	297,205	Overall		1.2
Ungar et al. 2001 [33]	339	GP	1995	3.6
		Respiratory specialist		2.1
***Adults***				
Boulet et al. 2008 [35]	514	Non-smoker	2004	43% had ≥1
	268	Former smoker		49% had ≥1
	108	Current smoker		47% had ≥1
Lemiere et al. 2007 [25]	351 (WEA: 145, OA: 206)	WRA	2001-2003	4.1(4.3)
		NWRA		1.2(1.7)
Sadatsafavi et al. 2010 [10]	158,516	Narrow asthma definition	1996-2000	1.86
		Broad asthma definition		3.85
Iron et al. 2003 [37]	230*	Asthma patients	1994/1995	4.3**
Sin et al. 2001 [44]	6,254	No ICS (elderly)	1992-1996	3.9(2.2)
		ICS (elderly)		4(2.2)
Anis et al. 2001 [38]	4,671	Appropriate use†	1995	14.9(15.9)
	763	Inappropriate use‡		16.7(19.3)
Anis et al. 2000 [34]	733	Physician visits in ED	1994-1995	1.0(1.3)
Blais et al. 2009 [18]	1264	BUD/FORM	2002-2004	7.5(7.4)
	1264	FP/SM		7.3(7)
***All ages***				
Gershon et al. 2007 [2]	NR	All-cause claims	1994/1995	13.2
			1995/1996	12.5
			1996/1997	12.0
			1997/1998	12.1
			1998/1999	11.9
			1999/2000	11.6
			2000/2001	11.5
			2001/2002	11.2

§ Per patient per year, mean (SD).

*Asthma patients, calculated as 6% of 3830 NPHS responders.

**Median.

†Appropriate use (low-dose SABA + high-dose ICS).

‡Inappropriate use (high-dose SABA + low-dose ICS).

BUD/FORM=budesonide/formoterol, FP/SM=fluticasone propionate/salmeterol, GP=general practitioner, ICS=inhaled corticosteroid, NR=not reported, NWRA=non-work-related asthma, WRA=work-related asthma.

More former or current smokers than non-smokers visited their physician [35], as did patients with work-related asthma vs. non-work-related asthma [25] and patients inappropriately using their asthma medication [38]. Among elderly patients, the rate of physician visits for asthma was not influenced by treatment with ICS [44].

Children in an Ontario-based study who were born in 1994 and diagnosed with asthma before age 6, and whose asthma persisted until age 11 (as determined by the presence of claims for physician and/or hospital visits between the ages of 6 and 11), had a higher rate of physician visits than those in remission (60 vs. 46.9 visits per 100 patients per year) [26].

#### ***Medication prescriptions***

Lynd et al. [40] reported that 27% of patients receive oral corticosteroids, 17% use no ICS, 47% receive less than 4 ICS canisters per year, 29% use 5 to 12 canisters, and 8% use more than 12 ICS canisters per year.

Based on available data, children received more prescriptions per patient per year than adults [11,21,38]. Patients with inappropriate use of asthma medications (ie, those who were non-adherent to guidelines recommended in the 1999 Canadian asthma consensus report and the National Heart, Lung and Blood Institute Guidelines for the Diagnosis and Management of Asthma) received more than double the number of prescriptions per patient per year (mean [SD] 7.5 [4.9]) compared with those who used asthma medication appropriately (mean [SD] 3.3 [1.9]) [38].

### Economic burden studies

#### ***Overview***

Ten studies evaluated the economic burden of asthma in Canada (5 cohort studies, 4 cross-sectional, and 1 economic analysis). Costs in the economic analysis were calculated for 1,350,871 persons, based on the 1998/1999 estimate that 57% of 2,389,085 persons aged ≥4 years had uncontrolled asthma.

More than 80% of these studies met the STROBE criteria for appropriate quality discussion. Most studies clearly reported the study design (90%), setting (100%), participants (90%), and statistical methods employed (70%). However, less than half reported on potential sources of bias and confounding factors or how missing data was handled and how loss to follow-up was addressed in both the methods and results sections or sensitivity analyses conducted. Most studies (60%) gave the source of funding and the role of the funders for their study (Table 1).

Asthma cases were identified using ICD codes or clearly stated diagnosis, retrospective physician visits, hospitalizations for asthma, and/or asthma medication prescriptions filled or patient self-report of asthma diagnosis or symptoms. There was available evidence on both the direct and indirect components of the economic burden of asthma in Canada. The overall burden varied based on whether studies reported costs from the perspective of an individual patient with asthma or costs at the population level. Few Canadian studies reported a cost per episode of acute asthma, and no studies reported the cost per patients overall. Five studies reported data on the direct costs of asthma at the patient-level. Three of these studies reported asthma costs per asthma patient [10,24,37], while 2 studies reported asthma costs per acute asthma episode [34,42]. Three studies reported population-level direct costs for asthma [10,24,42]. Study characteristics are presented in Table 7.

**Table 7:** Characteristics of economic burden studies included in the review

**Reference/Study period**	**Data source**	**Study objective**	**Inclusion criteria**	**Asthma definition**
***Retrospective cohort studies***				
Sadatsafavi et al. 2010 [10] 1996 - 2000	Administrative healthcare data	Determine direct medical costs of asthma-related healthcare in British Columbia	Aged 5 to 55 years	*Narrow definition:* ICD-9 code 493.x *Broad definition*: visits for an asthma-related diagnosis; hospitalizations with asthma among the discharge diagnoses
			≥4 asthma prescriptions in 1 year	
			≥1 asthma hospitalization	
			≥ 2 physician visits for asthma	
Malo et al. 2008 [46] 1988 - 2002	Administrative healthcare data,	Assess direct costs of CLI and CFI for OA and their association with selected variables	Subjects receiving compensation for OA	NR
To et al. 2008 [24] 1994 - 1998	DAD database from CIHI, OHIP records, RPDB database	Describe prevalence of asthma, all-cause mortality, physician visits, and hospitalizations for asthma and all causes; seasonal and geographical variation of healthcare utilization in children	Children aged 0–9 years	≥1 asthma hospitalization or 2 asthma OHIP claims within 3 years
***Prospective cohort studies***				
Ungar et al. 2001 [33] May - Oct 1995	Telephone interviews at 1, 3, and 6 months	Assess cost of asthma care at the patient level in children from the perspectives of society, the Ontario Ministry of Health, and the patient	Patients or caregivers filling prescriptions for bronchial inhalers	Probable asthma - a prescription for a bronchial inhaler medication in the last month (bronchodilator or corticosteroid) and reported experiencing shortness of breath, wheeze, or recurrent cough in the past
Anis et al. 2000 [34] Sept 1, 1994 - Aug 31 1995	2 hospital EDs in Saint John, NB; telephone interview for follow-up	Estimate average direct cost of illness for 4 cardiorespiratory conditions	ED visitors who completed follow-up interviews	ED visit records
***Cross-sectional studies***				
Kohen et al. 2010 [47] Fall 1998 and Spring 1999	NLSCY	Examine associations between asthma and school functioning	Individuals aged 7–15 years with complete data on the measures of interest	Past-year wheezing or whistling in the chest and regular use of inhalers
Boulet et al. 2008 [35] April - August 2004,	Telephone survey	Assess influence of current and former smoking on self-reported asthma control and healthcare use	Adults aged 18–54 years with physician-diagnosed asthma for ≥6 months	Patient report of physician-diagnosed asthma
Iron et al. 2003 [37] 1994/1995	CNPHS data linked with OHIP	Determine the association between demographics, access to care, SES, and need (comorbidities) with actual family physician costs	Survey respondents aged ≥25 years consenting to share HC number and responses with MOHLTC	Self-reported
Thanh et al. 2009 [48] 2005	CCHS	To estimate the cost of asthma-related productivity loss days due to absenteeism and presenteeism* in Alberta	Survey respondents aged 18–64 years	Patient report of an asthma diagnosis
***Health economic analysis***				
Seung et al. 2005 [42] 2004	NACRS at CIHI, OCCI, MOHLTC billing	Determine the use of urgent care resources and the annual costs of the uncontrolled asthmatic population in Canada	NR	ICD-9 code 493

* absenteeism=absent from work, presenteeism=at work but not fully functioning.

CCHS= Canadian Community Health Survey, CFI= compensation for functional impairment, CLI=compensation for loss of income, CNPHS=Canadian National Population Health Survey, HC=health card, MOHLTC=Ministry of Health and Long Term Care, NLSCY= National Longitudinal Survey of Children and Youth, OA=occupational asthma, OHIP=Ontario Health Insurance Plan, SES=socioeconomic status.

### Key findings on economic burden

All costs reported in this section are in 2011 Canadian dollars.

#### ***Patient-level direct costs***

Based on data from administrative databases in British Columbia, average total annual direct cost estimates in the general population ranged from $366.17 to $490.88 per asthma patient (Table 8) [10].

**Table 8:** Summary of studies that reported patient-level total direct costs for asthma

**Reference/Study period**	**Age group**	**Patient group**	**Average total annual cost per patient**	**Inflated 2011 $CAD**
***Retrospective cohort studies***				
Sadatsafavi et al. 2010 [10] Apr 1996 - Mar 2000	5-55 yrs	Narrow asthma definition	$331.15	$366.17
		Broad asthma definition	$443.93	$490.88
To et al. 2008 [24] 1994 - 1998	0-9 yrs	1994/1995	$535.9	$646.95
		1995/1996	$458.3	$553.27
		1996/1997	$392.6	$473.95
		1997/1998	$366.3	$442.20
		1998/1999	$332.9	$401.88
Ungar et al. 2001 [33] May - Oct 1995	0-14 yrs	Societal	$1,079	$1,410.17
		MOHLTC	$676	$883.48
		Patient	$76	$99.33

MOHLTC, Ministry of Health and Long Term Care.

Ungar and colleagues [33] estimated the total cost of asthma in children aged 0–14 years in Ontario to be $883.48 per child from the healthcare perspective (Table 8). Adjusted annual societal costs per patient (1995 Canadian dollars) ranged from $1,122 in children aged 4–14 years to $1,386 in children younger than 4 years. From the Ministry of Health perspective, adjusted annual costs per patient were $663 in children over 4 years and $904 in younger children. Adjusted annual costs from the patient perspective were $132 in children over 4 years and $129 in children under 4 years.

During the period from 1996 – 2000, average hospitalization costs ranged from $67.90 to $136.87 per patient per year in the general population in British Columbia (aged 5 to 55 years), depending on the definition used to categorize asthma-related hospitalizations [10]. The estimated average annual hospitalization cost for asthma in children was $682.21 per patient in Ontario [33].

Sadatsafavi et al. [10] reported that ED visits made by asthma patients in the general population could cost the healthcare system anywhere between $66.35 and $122.09 per visit, depending on the asthma definition used. The reported range of average costs for ED visit per acute asthma episode was $209.48 to $274.48 [10,33]. Ungar and colleagues [33] estimated the average annual cost for ED visits in children to be $15.68.

The average costs for physician visits per acute asthma episode were estimated to range from $31.72 [34] in an economic modeling study using prospectively collected resource utilization data (9/1/94 to 8/31/95) from hospital emergency department visitors to $31.91 in the economic analysis by Seung and Mittmann [42]. Average costs of $98.02 and $70.57 annually per pediatric patient were reported for family physician and specialist visits, respectively, in the prospective study by Ungar et al. [33]. Although the cost per respiratory specialist visit was higher than the cost per family physician visit ($105.40 vs. $51.40 for the first visit and $23.10 vs. $16.25 for an additional visit), nearly twice as many patients (271, or 80%) reported visiting a family physician, at an average annual use of 3.6, compared with a respiratory specialist (141, or 42% of patients), at an average annual use of 2.1 [33]. A study conducted in Ontario demonstrated that outpatient claim costs for persons with asthma exceeded those for persons without asthma by about $200 per person per year [2].

With regard to asthma medication prescriptions, the administrative database study from British Columbia estimated the average annual cost for asthma medication in the general population to be $231.92 per patient [10]. Ungar et al. [33] estimated the average annual costs for medication per patient in children to be $352.87 from the societal perspective and $86.26 (2011 $CAD) from the patient perspective. Estimated average medication costs per acute asthma episode ranged from $5.29 to $629.39 in these studies [10,33].

#### ***Population-level direct costs***

The 1998–1999 healthcare cost for asthmatic children in Ontario ($120 million, or $227.1 - $640.3 per child per year, depending on age group) was considerably higher than the total asthma cost for the general population (all ages) of British Columbia during the period 1996 – 2000 (~$41.8 million, or $331 per patient per year) (Table 9) [10,24].

**Table 9:** Summary of studies that reported population-level total direct costs for asthma

**Reference/Study period**	**Age group**	**Patient group**	**Total annual population cost**	**Inflated 2011 $CAD**
***Retrospective cohort studies***				
Sadatsafavi et al. 2010 [10] April 1996 - March 2000	5-55 yrs	Narrow asthma definition	$41,858,610	$46,285,583
		Broad asthma definition	$56,114,574	$62,049,260
To et al. 2008 [24] 1994 - 1998	0-9 yrs	1994/1995	$116,700,000	$140,882,165
		1995/1996	$114,800,000	$138,588,454
		1996/1997	$106,900,000	$129,051,443
		1997/1998	$105,300,000	$127,119,897
		1998/1999	$98,900,000	$119,393,711

MOHLTC, Ministry of Health and Long Term Care.

Based on data from administrative healthcare databases (April 1996 through March 2000), the total annual population-level asthma cost estimates in the general population in British Columbia ranged from ~ $46.3 million to $62.0 million , depending on the definition of asthma used [10]. Between ~8.5 million and ~17.2 million of that was spent on asthma-related hospitalizations, ~8.4 million to ~15.5 million on physician/ED visits, and ~15.4 million to ~29.3 million on asthma medications [10]. Medication costs represented the bulk (63.9%) of the total cost, hospitalizations/ED visits comprised 17.8%, and physician visits accounted for 18.3% of the total cost.

In Ontario, the total population-level costs for asthma in children aged 0–9 years ranged from ~ $140 million during 1994–1995 to ~ $120 million in 1998–1999 [24].

#### ***Patient-level indirect costs***

About 50% of children missed 1–3 days of school (47.6% in the group with low-severity asthma, 53.9% in those with moderate severity, and 50.6% in the severe asthma group), and 5.7% of the low severity, 5.3% of the moderate severity, and 9.1% of the severe asthma patients were absent for 7 or more days [47].

Malo et al. [46] evaluated a random sample of 8 to 10 accepted claims for occupational asthma per year from 1988 to 2002 in Quebec and found that the mean cost of compensation for loss of income (CLI) across the 15 years (not accounting for inflation) was $72,500 (median $40,700) and the mean cost of compensation for functional impairment (CFI) was $11,700 (median $7,600). Median CLI costs were significantly higher in men than women (69.9 vs 13.1), in workers aged ≥40 years versus those <40 years (90.1 vs 27.4), and in workers taking inhaled steroids at diagnosis (92 vs 52) and at reassessment (81 vs 35). Median CFI costs were significantly higher for individuals being treated with inhaled steroids at the time of diagnosis (14.0 vs 5.2) and reassessment (13 vs 6).

#### ***Population-level indirect costs***

In a population of ~1.5 million working-age individuals in Alberta with an asthma prevalence of 8.5%, the number of asthma-related productivity lost work days ranged from 441,728 to 533,363 in 1 year, at a cost of $78.1 to $94.4 million in lost productivity [48].

Ungar et al. [49] reported productivity loss days (PLD) without reporting actual indirect costs. They found that annual PLD varied from 12 in employed persons to 20 in students, 22 in homemakers, retirees and the unemployed, and 49 in disability pensioners. Annual PLDs increased with increasing disease severity.

### Humanistic burden studies

#### ***Overview***

Fourteen articles reporting results from 13 studies were retained for inclusion out of the 309 studies identified by the humanistic burden literature search. Two were cohort studies, 8 were cross-sectional, and 4 studies were RCTs. Only 1 of these was a pediatric study [50], which assessed the impact of asthma medication on children using the 3-domain Pediatric Asthma Quality of Life Questionnaire (PAQLQ). No studies were identified that reported utilities or QoL from a caregiver perspective. QoL assessments focused on subgroups of the asthma population, and studies had small numbers of participants. A variety of definitions were used to define asthma including clinical diagnosis, presence of symptoms, and positive inhalation tests. Characteristics of studies reporting on humanistic burden are detailed in Table 10.

**Table 10:** Characteristics of humanistic burden studies included in the review

**Reference/Study period**	**Design**	**Study objective**	**Inclusion criteria**	**Asthma definition**
Miedinger et al. 2011 [51] 2004 - 2006	Longitudinal study - subjects who claimed compensation for OA in Quebec	Examine association between clinical and socioeconomic variables and psychological and cost outcomes in patients with OA	Claimed compensation for OA at CSST, not exposed to offending allergens causing OA for ≥2 years	Workplace-associated respiratory symptoms and positive results in specific inhalation test
Lavoie et al. 2010 [52] NR	Prospective cohort, self-report questionnaires	Assess level of psychological distress and range of disease-relevant emotional and behavioural coping styles in patients with severe vs. moderate asthma	Patients aged 18–69 years recruited from 2 tertiary care outpatient asthma clinics	Standard ATS criteria; Severe asthma - received adequate therapy and verified treatment adherence, with patients meeting ATS major and minor criteria for severe asthma
Bacon et al. 2009 [53] Jun 2003 - Jan 2007	Cross-sectional study; patients administered questionnaires	Assess associations between adult SES (measured according to educational level) and asthma morbidity, including asthma control; asthma-related emergency health service use; asthma self-efficacy, and asthma-related QoL	Patients aged 18–75 years, recruited from outpatient asthma clinic of Hôpital du Sacré-Coeur de Montréal	Physician-diagnosed asthma - charted 20% fall in FEV1 after methacholine challenge and/or bronchodilator reversibility in FEV1 of ≥20% predicted; severity based on GINA guidelines (mild intermittent, mild persistent, moderate persistent, and severe persistent)
McTaggart-Cowan et al. 2008 [54] NR	Cross-sectional - self-administered questionnaire	Evaluate validity of HUI-3, EQ-5D, SF-6D, and AQL-5D to distinguish between different levels of asthma control	Patients aged 19–49 years,no other concurrent respiratory conditions	Self-reported, physician-diagnosed asthma
Rowe et al. 2007 [55] NR	RCT (double-blind) -structured telephone interviews	Examine effect of adding a LABA (salmeterol) to fixed dose of oral prednisone and ICS (fluticasone)	Patients aged 18–55 years, PEF of <80% predicted before treatment, discharged from ED	Clinically diagnosed acute asthma in ED; PEF of <80% predicted before treatment
Yacoub et al. 2007 [56] 2004 - 2006	Retrospective cohort study; questionnaire administered to subjects	Evaluate utility of adding assessment of airway inflammation to standard assessment of impairment in subjects with OA; to evaluate psychological and QoL impact of OA	Workers' Compensation Agency of Quebec claimants	OA claimants
Lavoie et al. 2006 [57] 2003 - 2005	Cross-sectional study; structured psychiatric interview	Evaluate relative impact of having a depressive and/or anxiety disorder on asthma control and QoL	Patients aged 18–75 years with primary diagnosis of asthma	Physician-diagnosed asthma - chart evidence of 20% fall in FEV1 after methacholine challenge and/or bronchodilator reversibility in FEV1 20% predicted; severity classified according to international GINA guidelines
Lavoie et al. 2006 [58] Jun 2003 to Apr 2004	Cross-sectional study; patients completed ACQ and AQLQ questionnaires	Assess BMI in a Canadian sample of asthma outpatients, and evaluate associations between BMI and levels of asthma severity, asthma control, and asthma-related QoL	Patients aged 18–75 years with primary diagnosis of asthma, fluency in either English or French	Physician diagnosed asthma - chart evidence of 20% fall in FEV1 after methacholine challenge and/or bronchodilator reversibility in FEV1 20% predicted; severity classified according to GINA guidelines
Lavoie et al. 2005 [52] NR	Cross-sectional study; patients completed ACQ and AQLQ questionnaires	Evaluate prevalence of psychiatric disorders in adult asthma patients and associations between psychiatric status, levels of asthma control, and asthma-related QoL	Patients aged 18–75 years with primary diagnosis of asthma, fluency in either English or French	Physician diagnosed asthma - confirmed by chart evidence of 20% fall in FEV1 after methacholine challenge and/or bronchodilator reversibility in FEV1 20% predicted; severity classified according to GINA guidelines
Mo et al. 2004 [59] 2000 - 2001	Cross-sectional study; HUI used to measure QoL	Measure HRQL of chronic disease and detect associations between HUI system and various chronic conditions	All household residents aged ≥12 years in all provinces and territories	NR
FitzGerald et al. 2000 [60]	RCT - AQLQ administered to assess QoL	Compare effectiveness of prednisone and budesonide on relapse rate	Patients aged 15–70 years, recruited after discharge from ED after acute asthma exacerbation	Asthma exacerbation - progressive increase in dyspnea and history of asthma as per ATS criteria
Williams et al. 2010 [61] Baseline to week 12	RCT AQLQ data from first 12 weeks of the GOAL study	Compare AQLQ data across 16 countries (17 languages)	Patients aged 12 to <80 years with ≥6-month history of asthma	NR
Miedinger et al. 2011 [51] 2004 to 2006	Cross-sectional study; participants completed validated French versions of QoL questionnaires	Assess correlation between asthma-specific QoL and levels of psychological distress and psychiatric disorders in patients with OA	Patients who claimed compensation for OA at CSST; no longer exposed to sensitizing agents ≥2 years	OA - asthma caused and maintained by conditions attributable to the occupational environment and not to stimuli encountered outside the workplace
Zimmerman et al. 2004 [50] 12-week study	RCT (double-blind); patients administered PAQLQ	Examine efficacy and safety of adding regular formoterol at 2 different doses to maintenance treatment with ICS in children with asthma not optimally treated by ICS alone	Patients aged 6–11 years with clinical diagnosis of asthma as per ATS criteria for ≥6 months; FEV1 50-90% of predicted normal; documented post-bronchodilator reversibility of ≥15%, ≥9% of predicted normal; treatment with regular ICS for ≥3 months before trial entry; asthma symptoms sufficient to suggest additional therapy may be needed; ability to use peak flow meter and Turbuhaler®, able to answer questions from PAQLQ; parent/guardian to complete daily diary	Clinical diagnosis of asthma defined according to ATS criteria; severe asthma exacerbation defined as asthma symptoms requiring oral corticosteroids or increase in dose of ICS as judged by the investigator

ACQ=Asthma Control Questionnaire; AQLQ=Asthma Quality of Life Questionnaire; AQL-5D=Asthma Quality of Life-5D ; ATS=American Thoracic Society; BMI=body mass index; BUD=budesonide; CSST=Commission de la Santé et de la Sécurité du Travail du Québec (Canadian Centre for Occupational Health and Safety); ED=emergency department; EQ-5D =EuroQoL 5-D ; FEV1=forced expiration volume in 1 second; GINA=Global Initiative for Asthma; GOAL=Gaining Optimal Asthma ControL (study); GSCs=glucocorticosteroid; HRQoL = health-related quality of life; HUI=health utilities index; ICS=inhaled corticosteroid; LABA=long-acting β-agonist; NR=not reported; OA=occupational asthma; PAQLQ=Pediatric Asthma Quality of Life Questionnaire; PEF=peak expiratory flow; PRED=prednisone; PRIME-MD=Primary Care Evaluation of Mental Disorders; PSI=Psychiatric Symptom Index; QoL = quality of life; RCT=randomised controlled trial; SES=socioeconomic status; SF-6D=Short-Form 6D; SGRQ=St-Georges Respiratory Questionnaire.

Overall, most studies on humanistic burden met good reporting quality standards in accordance with STROBE criteria (Table 1). However, less than half of the studies reported how missing data and loss to follow-up was handled or sensitivity or other analyses performed. Most studies also met the STROBE criteria for appropriate quality discussion (80%) and reported information on study funding (80%).

The effect of psychiatric disorders on asthma control and QoL in adults was examined in 2 studies [57,58]. Another 2 studies examined QoL by asthma severity and chronicity [54,62]. Eleven studies used the 32-item AQLQ to assess the impact of asthma on patients’ QoL [51-56,58-63]. Other tools that were used to measure the humanistic burden of asthma were the AQL-5D, the EQ-5D, the SF-6D, the Health Utilities Index (HUI-3), the Asthma Control Questionnaire (ACQ), and the 8-question St Georges Respiratory Questionnaire (SGRQ).

### Key findings on humanistic burden

Depression and anxiety were prevalent among asthma patients and were associated with worse asthma control and quality of life (QoL) [52]. Yacoub et al. reported a 50% prevalence of anxiety and/or depression among 40 subjects with occupational asthma [56]. In a study conducted by Lavoie et al., 31% of 504 adults with physician-diagnosed asthma met the diagnostic criteria for 1 or more psychiatric diagnoses [57]. A study specifically looking at occupational asthma also found that psychological distress and psychiatric disorders including depression, anxiety, and dysthymia were associated with impaired QoL [63].

As one would expect, QoL became progressively worse as disease severity increased [54,62]. Furthermore, QoL was lower in asthma patients who had at least 1 other chronic disease compared to those who had no other chronic disease [54].

A study of 504 consecutive adults with physician-diagnosed asthma reported that depressive and anxiety disorders were both independently associated with decreased health-related QoL (as measured by AQLQ scores), but only depressive disorders were independently associated with worse asthma control (as measured by ACQ scores) [57]. Interestingly, having both depressive and anxiety disorders did not increase the risk for worse asthma control or decreased QoL [57]. According to the study authors, this finding suggests that there is no incremental risk associated with having both a depressive disorder and an anxiety disorder on asthma control and QoL. The researchers also noted that the lack of an independent association between anxiety disorders and asthma control may be due to the fact that patients with anxiety disorder are more inclined to self-monitor their symptoms, and are thus more likely than depressed patients to detect asthma symptoms and seek intervention.

Lavoie et al. [58] studied the association between clinical measures of asthma morbidity and body mass index (BMI), and found that patients with higher BMI scores had worse asthma control and poorer QoL (i.e., higher ACQ and lower AQLQ scores), independent of age, gender, and asthma severity. However, BMI was not associated with asthma severity.

## Discussion

This review is the first to summarize the literature encompassing not only the clinical and economic burden of asthma, but also the humanistic burden of asthma in Canada. This systematic review confirms that the burden associated with asthma is substantial, and will undoubtedly become more pronounced as the asthma prevalence increases in Canada. The asthma burden as it is known today can likely be decreased by the development and implementation of innovative treatment strategies in the management of this disease.

A considerable body of literature was included in this systematic review (33 articles for the clinical and economic burden and 14 for the humanistic burden).The reviewed literature suggested that the healthcare resource utilization in asthma varied greatly in Canada by age group and type of treatment used. The substantial clinical burden was reflected by high rates of hospitalizations, ED and physician visits, and medication use. Lower rates of ED visits and hospitalizations, as well as reduced deaths, were observed among ICS users compared with non-users (except among the elderly), but these reductions were not as pronounced in patients who had experienced recent asthma exacerbations.

We collected evidence on both the direct and indirect components of the economic burden of asthma in Canada. The overall burden varied based on whether studies reported costs from the perspective of an individual patient with asthma or costs at the population level. Reported estimates for patient-level total direct costs, inflated to 2011 Canadian dollars, ranged from $99.33 per patient in a cohort of children aged 0–14 years in Ontario (May – October 1995) [33] to $646.95 per patient in a cohort of children aged 0–9 years, also in Ontario (1994/1995) [24]. Reported estimates for population-level total direct costs, inflated to 2011 Canadian dollars, ranged from $46,285,583 for patients aged 5–55 years in British Columbia (April 1996 – March 2000) [10] to $140,882,165 for patients aged 0–9 years in Ontario (1994–1998) [24]. Few Canadian studies reported a cost per episode of acute asthma.

Fourteen studies assessed the impact of asthma on the QoL of patients; however, only 4 reported on QoL of children with asthma, which represents a significant knowledge gap. For the most part, QoL assessments focused on subgroups of the asthma population and studies had small numbers of participants. Asthma was associated with depression and/or anxiety in several studies.

As noted above, these research studies vary considerably in terms of geographic region of study, characteristics of patient populations, study methodologies, and definitions of asthma used, which presents a significant challenge in drawing definitive conclusions from our study. Furthermore, unique findings reported in single studies have yet to be confirmed or refuted by subsequent research. Thus, in our review, results are presented as reported, but no consensus can be reached on the rates of resource utilization among asthmatic patients, asthma-related costs, or the degree of QoL impairment among individuals with asthma.Our study suggests that there is a significant knowledge gap in understanding the comprehensive burden of asthma across Canada.

Nevertheless, the high rates of healthcare resource utilization observed among patients with asthma during this review revealed only the tip of the iceberg. The economic burden is noteworthy, with direct costs – particularly those related to hospitalizations and physician/ED visits – representing the highest proportion of asthma-related costs. The indirect costs mainly due to time loss from work, productivity loss, functional impairment and caregiver time also add to this significant burden. Although there is a paucity of research on the humanistic burden of asthma in Canada, the few studies included in this review indicate that QoL is unquestionably diminished in asthmatic patients and that there is a high prevalence of psychological distress and psychiatric disorders among patients with asthma. Notable knowledge gaps on the humanistic burden of asthma are the lack of QoL assessments in children and caregivers, as well as quantifying the asthma-attributable burden in this patient population.

This systematic review provides a holistic overview of the burden of asthma in Canada, detailing the direct and indirect costs, the key drivers of healthcare resource utilization, and the impact of asthma on patients’ quality of life - information that cannot be inferred from clinical measures. This information can be of value to payers, policy makers and healthcare providers in making decisions pertaining to the management and treatment of asthma.

For example, knowing that depression is often associated with asthma and that its severity and asthma control are intertwined, it might be useful to have psychologists/psychiatric healthcare professionals on the disease management team from the time of asthma diagnosis. Also, findings that BMI levels and asthma control and QoL are related, can lead to adding interventional measures to the treatment strategy.

As far as treatment options go, the use of inhaled corticosteroids was noted in many of the reviewed articles to be associated with lower rates of ED visits and hospitalizations; therefore recommending the appropriate use of medications (low-dose SABA plus high-dose ICS) should be emphasized.

More research in Canada is needed to add to the holistic picture of the impact of this disorder on the lives of patients, their families, and caregivers. Furthermore, much remains to be learned about the optimal use of the currently available treatments, how to combine them for maximal benefit, and how to incorporate new drugs in development into existing treatment regimens.

### Limitations

All literature reviews are limited by the publication bias of the articles that are available. We acknowledge the fact that studies identifying a significant burden of asthma may likely be published than the ones reporting a low burden. The articles in this review are limited to the English language, and publication constraints were placed on articles identified by the search with studies limited to those published since 2000. Spatial restrictions were also applied, limiting studies to Canada. Studies employed a variety of defining criteria for asthma (from patient self-report to ICD-codes, from physician-recorded diagnosis to discharge diagnosis combined with medication use), and these definitions were not reconciled in this review. This may have led to underreporting or overreporting of certain outcomes. Results were analyzed as reported, but direct comparisons between studies are lacking, due to the high heterogeneity of methodological approaches.

In spite of these limitations, this review was systematic in nature and summarizes all available and relevant data published since 2000, thus providing a better understanding of the literature with respect to the clinical, economic, and humanistic burden of asthma.

## Conclusions

The information contained within this study provides a comprehensive overview of the burden of asthma in Canada. Moreover, our study identifies several key knowledge gaps in understanding this area. As new therapies for asthma become available, health technology assessments will become increasingly important not only as it pertain to amendments to clinical practice guidelines but also with regard to formulating reimbursement decisions. Our study summarizes information that can prove important for physicians, healthcare authorities, and government officials involved in the treatment selection and development of disease management guidelines for asthma.

## Appendix

The Appendix tables present the literature search strategies used to retrieve articles reporting on the clinical and economic burden (Table 11) and humanistic burden (Table 12) of asthma. The strategies were applied to the Medline. EmBase and EMCare databases.

**Table 11:** Clinical and economic burden search strategy

**Medline (1996 to present)**		
1	Asthma[MeSH] OR Asthma [Title,abstract]	71642
2	hospitalisation[MeSH] OR cost of illness[MeSH] OR absenteeism OR ambulatory care/Economics[MeSH] OR drug costs[MeSH] OR emergency medical services/Economics[EMTREE] OR healthcare costs[MeSH] OR nursing services/Economics[MeSH] OR physicians/Economics[MeSH]	77559
3	(burden OR clinical impact OR hospitalisation OR utilization OR burden of illness OR cost$1 OR cost of illness OR utilization OR nursing cost$1 OR physician cost$1 OR physician visit$1).TI,AB.	354392
4	1 AND (2 OR 3)	6208
5	canada OR canadian OR alberta OR british columbia OR manitoba OR new brunswick OR newfoundland NEXT labrador OR northwest territories OR nova scotia OR nunavut OR ontario OR prince edward island OR quebec OR saskatchewan OR yukon NEXT territory	462814
6	4 AND 5 AND LG=English AND HUMAN=YES	430
7	Publication Type=RANDOMIZED CONTROLLED TRIAL	223783
8	6 NOT 7	398
9	limit set 8 YEAR > 1999	324
EmBase (1992 to present)		
10	Asthma[EMTREE] OR Asthma[Title,abstract]	100645
11	hospitalisation[EMTREE] OR cost of illness[EMTREE] OR cost[EMTREE] OR absenteeism[EMTREE] OR drug cost[EMTREE] OR healthcare cost[EMTREE] OR nursing cost[EMTREE]	348772
12	(burden OR clinical impact OR hospitalisation OR utilization OR burden of illness OR cost$1 OR cost of illness OR utilization OR nursing cost$1 OR physician cost$1 OR physician visit$1)[Title,abstract]	381230
13	10 AND (11 OR 12)	10735
14	canada OR canadian OR alberta OR british columbia OR manitoba OR new brunswick OR newfoundland NEXT labrador OR northwest territories OR nova scotia OR nunavut OR ontario OR prince edward island OR quebec OR saskatchewan OR yukon NEXT territory	462680
15	13 AND 14 AND LG=English AND HUMAN=YES	654
16	Randomized Controlled Trial[EMTREE] OR Randomized Controlled Trial Topic[EMTREE]	249284
17	15 NOT 16	596
18	limit set 17 YEAR > 1999	515
EMCare		
19	Asthma[EMTREE] OR Asthma[Title,abstract]	28554
20	hospitalisation[EMTREE] OR cost of illness[EMTREE] OR cost[EMTREE] OR absenteeism[EMTREE] OR drug cost[EMTREE] OR healthcare cost[EMTREE] OR nursing cost[EMTREE]	152470
21	(burden OR clinical impact OR hospitalisation OR utilization OR burden of illness OR cost$1 OR cost of illness OR utilization OR nursing cost$1 OR physician cost$1 ORphysician visit$1)[Title,abstract]	156234
22	19 AND (20 OR 21)	4228
23	canada OR canadian OR alberta OR british columbia OR manitoba OR new brunswick OR newfoundland NEXT labrador OR northwest territories OR nova scotia OR nunavut OR ontario OR prince edward island OR quebec OR saskatchewan OR yukon NEXT territory	174145
24	22 AND 23 AND LG=EN	312
25	Randomized Controlled Trial[EMTREE] OR Randomized Controlled Trial[EMTREE]	82273
26	24 NOT 25	278
27	limit set 26 YEAR > 1999	222
**Medline, EmBase and EMCare combined**		
28	combined sets 9, 18, 27	1061
29	dropped duplicates from 28	486
30	unique records from 28	575
31	split set 30	320 Medline
32	split set 30	234 EmBase
33	split set 30	21 EmCare

**Table 12:** Humanistic burden search strategy

**Medline**		
1	Asthma[MeSH] OR Asthma[Title,Abstract]	71642
2	Sickness impact profile[MeSH] OR quality of life[MeSH] OR patient satisfaction[MeSH]	121478
3	(quality of life OR QoL OR patient reported outcome$1 OR patient satisfaction OR emotional satisfaction OR patient dissatisfaction OR patient response OR gratification OR treatment satisfaction OR disability rate$1 OR health related quality of life OR HRQoL OR utilities) [Title,Abstract]	119368
4	1 AND (2 OR 3)	3035
5	canada OR canadian OR alberta OR british columbia OR manitoba OR new brunswick OR newfoundland NEXTlabrador OR northwest territories OR nova scotia OR nunavut OR ontario OR prince edward island OR quebec OR saskatchewan OR yukon NEXT territory	462814
6	4 AND 5 AND LG=English AND HUMAN=YES	172
7	limit set 6 YEAR > 1999	141
**EmBase**		
8	Asthma[EMTREE] OR Asthma[Title,Abstract]	100645
9	Sickness impact profile[EMTREE] OR quality of life[EMTREE] OR patient satisfaction[EMTREE]	199618
10	(quality of life OR QoL OR patient reported outcome$1 OR patient satisfaction OR emotional satisfaction OR patient dissatisfaction OR patient response OR gratification OR treatment satisfaction OR disability rate$1 OR health related quality of life OR HRQoL OR utilities) [Title,Abstract]	124979
11	8 AND (9 OR 10)	5651
12	canada OR canadian OR alberta OR british columbia OR manitoba OR new brunswick OR newfoundland NEXT labrador OR northwest territories OR nova scotia OR nunavut OR Ontario OR prince edward island OR quebec OR saskatchewan OR yukon NEXT territory	462680
13	11 AND 12 AND LG=English AND HUMAN=YES	306
14	limit set 13 YEAR > 1999	267
**EMCare**		
15	Asthma[EMTREE] OR Asthma[Title,Abstract]	28554
16	Sickness impact profile[EMTREE] OR quality of life[EMTREE] OR patient satisfaction[EMTREE]	94595
17	(quality of life OR QoL OR patient reported outcome$1 OR patient satisfaction OR emotional satisfaction OR patient dissatisfaction OR patient response OR gratification OR treatment satisfaction OR disability rate$1 OR health related quality of life OR HRQoL OR utilities) [Title,Abstract]	48206
18	15 AND (16 OR 17)	2178
19	canada OR canadian OR alberta OR british columbia OR manitoba OR new brunswick OR newfoundland NEXT labrador OR northwest territories OR nova scotia OR nunavut OR Ontario OR prince edward island OR quebec OR saskatchewan OR yukon NEXT territory	174145
20	18 AND 19 AND LG=English	137
21	limit set 20 YEAR > 1999	111
**Medline, EmBase and EMCare combined**		
22	combined sets 7, 14, 21	519
23	dropped duplicates from 22	207
24	unique records from 22	312
25	split set 24	141 Medline
26	split set 24	158 EmBase
27	split set 24	13 EMCare

## Competing interests

ASI and APS are employees in the Medical Division of GlaxoSmithKline Inc., Canada. ASI is also an assistant professor (part-time) in the Department of Clinical Epidemiology and Biostatistics at McMaster University, Hamilton, Ontario, Canada. MM is an employee of Optum. ZS was an employee of GSK at the time of the research and analyses of this project. ZS is currently an employee of Sanofi.

## Authors’ contributions

All authors contributed to the design and protocol of the study. ASI, MM and APS identified and reviewed the literature to include in the systematic review. ZS provided the medical interpretation of the data. APS coordinated the review and finalization of the manuscript. All authors reviewed the results of the analyses and contributed to, read and approved the final manuscript.

## Pre-publication history

The pre-publication history for this paper can be accessed here:

http://www.biomedcentral.com/1471-2466/13/70/prepub
